# Endoscopic treatment of vesicoureteral reflux: Current status

**DOI:** 10.4103/0970-1591.45534

**Published:** 2009

**Authors:** Göran Läckgren

**Affiliations:** Section of Urology, University Children's Hospital, Uppsala, Sweden

**Keywords:** Children, treatment, vesicoureteral reflux

## Abstract

Vesicoureteral reflux (VUR) affects around 1% of all children. It carries an increased risk of febrile urinary tract infections (UTIs) and is associated with impaired renal function. Endoscopic treatment with NASHA/Dx gel (dextranomer microspheres in a stabilized hyaluronic acid-based gel of nonanimal origin) is minimally invasive, well tolerated and provides cure rates approaching those of open surgery: ∼90% in several studies. It has also been shown to be effective in a variety of ‘complicated’ cases. Endoscopic treatment is therefore considered preferable to open surgery and long-term antibiotic prophylaxis. Nontreatment of VUR is being discussed as an alternative option, whereby children are treated with antibiotics only when UTIs occur. Considering all the available evidence, however, active intervention with endoscopic treatment remains preferable. A new approach to managing VUR may nevertheless be considered, with treatment decisions based not only on the grade of reflux, but also factors such as age, sex, renal scarring, and bladder dysfunction. Open surgery would be reserved for use only in the ^∼^10% of children not responding to endoscopic treatment, and patients with refluxing primary megaureter.

Vesicoureteral reflux (VUR) is characterized by backflow of urine from the bladder up the ureter toward the kidneys. It is the most common pediatric anomaly of the urinary tract, affecting around 1% of all children.[1] Urinary tract infections (UTIs) occurring in children with VUR are more likely (than in children without VUR) to reach the upper urinary tract and develop into pyelonephritis which, in turn, is associated with febrile illness and a possible risk of renal scarring.[2,3] Correspondingly, permanent renal damage has been reported in 73% of children with recurrent UTIs.[3] Therefore, for most patients with VUR, treatment is recommended to avoid the risks associated with febrile UTIs.[2,4]

Vesicoureteral reflux generally resolves with increasing age. However, reflux often persists for a number of years and, in some cases, does not resolve and may persist into adulthood. For example, among children with primary grade III-IV reflux, reflux (any grade) was found to persist for at least 5 years in 85% of cases (reflux of grade ≥ III persisted in 48%).[5] In the same study, at 10 years, reflux of any grade was present in 48% of cases (grade ≥ III in 23%). Thus, our problem today is how we should manage those patients with persistent reflux - should we treat them or not? It has been shown that adults with persistent reflux and scarring may run the risk of further pyelonephritis, progressive renal disease, and hypertension.[6,7] There are several related questions: ‘does the risk of scarring and hypertension persist without reflux?,’ ‘is there an increased risk for women with reflux during pregnancy?,’ and ‘which patients are most likely to develop complications relating to persistent reflux?’

Endoscopic treatment involves submucosal injection of a bulking agent into the bladder wall below the ureteral orifice, or within the ureteral tunnel, to provide tissue augmentation. The other treatment options for VUR are antibiotic prophylaxis and open surgery (ureteral reimplantation). Endoscopic treatment is a minimally invasive procedure offering a high chance of cure through a single intervention. Open surgery, while also offering a high chance of cure, is a more invasive (and traumatic) procedure for children; it carries an increased risk of significant complications and an overnight hospital stay is usually required. Antibiotic prophylaxis is administered to prevent UTIs, with the intention of ceasing treatment once the condition has resolved. Although this approach avoids surgical intervention, there remains a risk of UTIs among children undergoing such treatment (related to poor compliance or resistant bacteria), and long-term antibiotic use is controversial due to the potential for increased resistance rates among bacteria in the community. Comparative appraisal of these treatment options has led to the conclusion that endoscopic treatment is generally the most favorable of these options for managing VUR.[2,8] Moreover, parents of children with VUR are very likely to express a preference for endoscopic treatment after all the options have been explained to them (in a survey, 80% expressed a preference for endoscopic treatment, compared with 5% for antibiotic prophylaxis, and 2% for open surgery).[9]

The choice of injectable agent is a key to the success of endoscopic treatment. To ensure safety and long-term efficacy, the ideal injectable agent should be biocompatible. The risk of new renal scarring is the greatest among infants and young children aged under 5 years.[10] Therefore, the bolus created using an injectable agent should persist for a minimum of 5 years. NASHA/Dx gel - dextranomer microspheres in a stabilized hyaluronic acid-based gel of nonanimal origin - was developed specifically for endoscopic treatment. Preclinical studies demonstrated the biocompatibility of NASHA/Dx gel, together with a lack of potential for migration from the injection site.[11,12] Both constituents of NASHA/Dx gel are biodegradable polysaccharides, ensuring that this material cannot accumulate permanently within the body. Injected NASHA/Dx gel becomes infiltrated with endogenous connective tissue, and follow-up studies (both preclinical and clinical) have shown that the bolus persists for at least 3 years with no fibrosis or aggressive granulomatous reaction spreading to adjacent tissue.[13,14] Long-term data indicate that the clinical effect of NASHA/Dx gel lasts much longer than this, over a period of 7-12 years, and that only 3% of patients experienced a febrile UTI over that period.[15] The consistency of NASHA/Dx gel is excellent for submucosal injection as it can be administered using finger pressure only, with no need for an injection gun. NASHA/Dx gel has become the dominant injectable agent for endoscopic treatment of VUR and, accordingly, it will be the primary injectable discussed in this review.

The aim of this article is to summarize our current knowledge and experience of endoscopic treatment of VUR, and discuss the role of this therapy relative to other treatment options. In addition, the classification of VUR will be examined, as it is becoming apparent that treatment decisions should be based not just on the grade of reflux, but also aspects such as bladder function and renal status.

## OUTCOMES FOLLOWING ENDOSCOPIC INJECTION

### Efficacy (VUR resolution)

The first major study of endoscopic treatment with NASHA/Dx gel involved 228 children with VUR; ureters with reflux grade II-V were treated with up to three NASHA/Dx gel injection procedures. All patients were scheduled for voiding cystourethrogram (VCUG) investigation 3-12 months after endoscopic treatment. The study included long-term clinical follow-up (mean duration 5 years), and a late VCUG (median 3 years after the last endoscopic treatment) was performed in 49 children. At their last VCUG, 68% of patients had reflux grade 0 or I and were therefore considered cured.[15,16]

More recent studies have reported much higher overall success rates with endoscopic treatment, approaching those seen with open surgery (in the region of 90%), after up to 12 months' follow-up.[17–19] The VUR cure rate has also been shown in a randomized, prospective study to be significantly higher following NASHA/Dx gel than with 12 months of antibiotic prophylaxis.[20] There is an apparent learning curve with the treatment procedure: in one study, success rates increased from 60% for the first 20 of 134 patients treated, to 80% for the last 20 cases.[21]

Many patients respond to a single treatment: in two studies, around three-quarters of patients were cured (reflux grade 0) at 3 months[21,22] and, in another study, reflux was corrected in 86% of ureters at 3-12 months' follow-up.[17] Nevertheless, in patients not responding to the first procedure, repeat endoscopic injection is viable. Returning to data from the first large study of NASHA/Dx gel, the response rate following the first injection was 54%; for the second injection it was 43%; and for the third it was 50%.[23]

Perhaps most importantly, the response to NASHA/Dx gel does not deteriorate over time. In one study, of ureters free from reflux at 3-12 months, 96% remained free from dilating reflux (i.e. they did not have reflux grade ≥ III) at 2-5 years' follow-up.[23] These data are consistent with the histopathological data indicating long-term persistence of the bolus created by NASHA/Dx gel endoscopic treatment.[14]

Cure rates may be optimized by using the hydrodistention-implantation technique (HIT), a modified version of the original subureteral transurethral injection (STING) technique. The more recent technique involves injection of NASHA/Dx gel into the mucosa of the ureteral tunnel, while a pressured stream of irrigation fluid is directed into the ureter to keep it open. VUR cure rates have been reported to be significantly greater among children treated via the HIT (89%), compared with the standard STING method (71%).[19]

**Figure 1:**
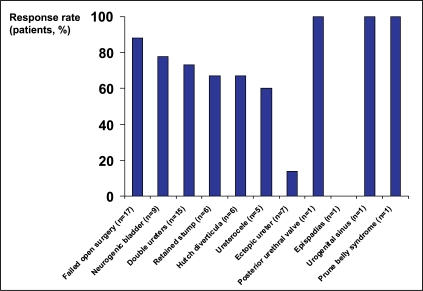
Success rates (grade 0) following endoscopic treatment with NASHA/Dx gel in complicated cases, 3 months posttreatment.[26]

Several studies have shown that endoscopic treatment is effective in VUR patients with complicating factors. The cure rate following NASHA/Dx gel treatment in children with VUR and duplicated ureters was 63%, while for those with a small kidney (one kidney contributing 10-35% of total renal function) the cure rate was 70%.[24] The percentage of children in these groups undergoing subsequent open surgery was 25% and 23%, respectively. More recently, clinical experience in children with VUR and bladder dysfunction was documented.[25] After 1-3 endoscopic treatments, 83% of patients had been cured, and bladder dysfunction had resolved in 59%. One study included children with a variety of different complications, including failure to respond to open surgery, duplicated ureters, neurogenic bladder, and ectopic ureters.[26] While the numbers of patients in some of these subgroups were small, success rates were high, indicating that endoscopic treatment should be considered as an option in children with a wide range of complications [Figure 1].

Recently, increasing evidence has been emerging to support the use of endoscopic treatment in patients with high-grade reflux. One retrospective study examined the results specifically from children with grade V reflux, and found that VUR was eliminated (grade 0) in 53% of ureters after a single procedure.[27] Following up to two repeat procedures, reflux persisted in only 3.6% of ureters. In a prospective study by the same authors, a resolution rate of 79.9% was observed following a single endoscopic treatment with NASHA/Dx gel, in a cohort of patients including many ureters (*n* = 248) with grade IV reflux and some (*n* = 17) with grade V reflux.[28]

## POSTTREATMENT INCIDENCE OF URINARY TRACT INFECTIONS

Low rates of UTI have been observed following endoscopic treatment with NASHA/Dx gel. For example, only 6/179 patients (3.4%) had proven pyelonephritis during long-term posttreatment follow-up over 7-12 years.[15] In another large cohort of patients (*n* = 276), albeit with a shorter follow-up period, UTIs after endoscopic treatment were reported in only three children (1%), although pyelonephritis was reported as a posttreatment complication in one case.[28] A third study showed that the mean incidence of UTIs, among those patients reporting any UTI, fell fivefold after endoscopic treatment, compared with the pretreatment incidence (0.12 vs. 0.68 UTIs/year, *P* = 0.001).[22] The overall reduction in UTI frequency was considerably greater (approximately 33-fold), as the percentage of patients experiencing UTIs reduced from 75% to 13%.

These results compare favorably with the incidence of UTIs following ureteral reimplantation or during antibiotic prophylaxis.[29,30] In the European arm of the International Reflux Study, after 5 years' follow-up, febrile UTI was reported to occur in 33 of 150 patients (21%) managed conservatively, and in 15 of 147 patients (10%) of children who underwent ureteral reimplantation.[29] In the USA arm, febrile UTI was observed in 22% (15 of 68 patients) and 8% (5 of 64 patients) of children managed medically and surgically, respectively.[30]

Possible reasons for this apparent advantage with endoscopic treatment include the likelihood that it is effective for both VUR and bladder dysfunction,[25] the fact that endoscopic treatment provides rapid cure of VUR, and the minimally invasive nature of the procedure. There is also evidence that surgery may lead to long-term alteration of bladder motility patterns.[31]

## SAFETY AND TOLERABILITY

A number of studies have shown that almost all children treated endoscopically do not experience any significant complications or adverse events. No complications were observed in the first large-scale study of NASHA/Dx gel (310 procedures in 228 children),[16] and this was also true of a subsequent study involving 113 children.[17] In another study, the only adverse event was mild, transient flank pain, affecting 2/120 patients;[18] flank pain or emesis affected 4% of children in a further study.[19] There have been isolated cases of ureteral obstruction or hydronephrosis following endoscopic treatment with NASHA/Dx gel,[32] but the overall incidence of this has been estimated to be <0.7%.[33] Ureteral obstruction may treated using a stent, with resolution likely within 7-30 days. A further possible complication following endoscopic treatment is de novo reflux in the contralateral (untreated) ureter, among children treated for unilateral reflux. This is a recognized complication of both surgical procedures and endoscopic injection. In one study, contralateral reflux was reported to affect 6/134 patients (4.5%).[21] A more recent study showed the incidence of contralateral reflux to be 10.1%, and the grade of reflux was I-II in 49% of these cases.[34] The authors concluded that prophylactic treatment of nonrefluxing contralateral ureters (during the treatment procedure for unilateral reflux) is not warranted, due to the low grade and low incidence of *de novo* reflux.

Histological findings confirm the lack of adverse reactions to NASHA/Dx gel. In a study published in 2003, 13 patients who responded poorly to endoscopic treatment underwent open surgery to cure their reflux.[14] The implanted NASHA/Dx gel and surrounding tissue were resected, and histological analysis showed a mild, granulomatous reaction of the giant cell type, as expected for the implantation of any foreign material. Thus, both the clinical and histological data for NASHA/Dx gel are consistent with the biocompatible nature of this material and the initial, favorable preclinical data.[11]

## COST-EFFECTIVENESS OF ENDOSCOPIC TREATMENT

Several studies have shown that endoscopic injection is more cost-effective than open surgery. The first such study showed the overall cost of endoscopic treatment was 25,000-28,000 SEK, compared with 65,000-90,000 for open surgery.[35] Endoscopic injection was also shown in this study to be more cost-effective than antibiotic prophylaxis. A subsequent study calculated that the overall cost of treating VUR by open surgery was 6640 USD, compared with 5522 USD when endoscopic treatment was used in place of open surgery.[36] A more recent economic analysis also concluded that endoscopic treatment ‘may be more cost-effective than ureteral reimplantation for children who meet standard criteria for surgical therapy, especially for lower grades of reflux.’[37]

Cost-saving strategies can also be employed when administering endoscopic treatment. First, the injection volume can be limited (1.0-1.2 ml can provide maximum efficacy) and, second, unnecessary follow-up investigations can be eliminated (a follow-up VCUG examination is unnecessary after a positive response to treatment has been demonstrated).

## APPROACHES TO MANAGING VUR

### Previously published treatment algorithm

**Figure 2:**
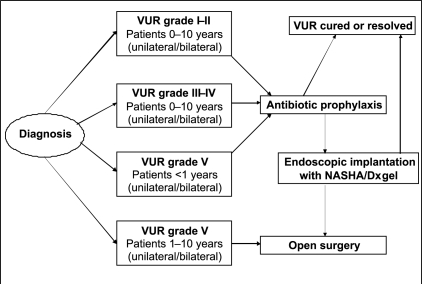
Treatment algorithm for children with vesicoureteral reflux (VUR), as published in 2003[4] (initial version published in 2002[8]). This figure is reproduced with permission from the publishers American Urological Association and Current Medicine Group, LLC; please see reference list for details of the source publications

Several years ago, a treatment algorithm for VUR was proposed, initially in 2002[8] and subsequently in 2003 as part of an American Urological Association update series [Figure 2].[4] In accordance with clinical data available at the time, endoscopic treatment was recommended as first-line active treatment for children with persistent reflux. Open surgery was reserved for children failing endoscopic treatment, or considered to be at high risk of kidney damage (reflux grade V in children aged 1-10 years). Thus, only a small proportion of children with VUR would undergo open surgery. A very similar treatment strategy was advocated around the same time by other European authors.[9]

Treatment algorithms generally require patients to be classified for determining therapy and, in the case of VUR, reflux grade has been the primary means of classification. The VUR grading system is dependent on the extent of reflux and the degree of structural abnormality (ureteral dilation). This approach does not account for factors such as age, sex, renal scarring, bladder dysfunction, and whether reflux peaks during bladder filling or voiding. Such factors are now understood to have significant bearing on the patient's prognosis, and a revised approach to the classification of VUR would be valuable. Indeed, it is ironic that we became so expert at surgically correcting VUR before understanding much of the natural history and true clinical significance of the condition.

## IS THERE A PLACE FOR NONTREATMENT OF VUR?

Recently, the possibility of not actively treating VUR has been proposed, whereby antibiotic therapy is administered only when UTIs occur.[38] Arguments in favor of this approach include the high-spontaneous cure rate of VUR, lack of reduction in renal complications associated with treatment, and the likelihood that VUR is caused by bladder dysfunction (treat the bladder instead).[38,39] On the other hand, there are several strong arguments against nontreatment. Cure of VUR is associated with a reduction in febrile UTI/pyelonephritis,[39] thereby decreasing overall morbidity. VUR persists for at least 5-10 years in many cases^5^ (particularly high-grade reflux associated with scarred kidneys[40]), placing susceptible kidneys at potential risk of progressive renal damage. Also, despite the widely held belief that bladder dysfunction causes VUR, there is now evidence that VUR may cause bladder dysfunction (it is of course possible that both of these scenarios may be true to some extent, with variation between cases).[25] In patients where VUR causes bladder dysfunction, problems experienced before resolution of reflux are exacerbated, and VUR with bladder dysfunction and recurrent UTI is difficult to treat conservatively. Children with VUR who are treated successfully have no need for further monitoring, in contrast to children with continuing VUR who likely require an increased number of VCUG investigations, which are traumatic for children and carry a risk of morbidity.[2] Finally, there is a possibility that high-grade VUR in infants may delay maturation of bladder function.

On balance, active treatment appears to remain the best option for the majority of children with persistent VUR - depending on factors such as the grade of reflux, renal status, and bladder function.

## CURRENT PRACTICE

There is a need to determine treatment not only according to reflux grade, but also renal scarring, bladder dysfunction, and whether reflux peaks during bladder filling or voiding, as these factors affect the risk of infection, risk of scarring, and likelihood of resolution. On this basis, endoscopic treatment may be considered as first-line therapy for all children with persistent reflux requiring intervention (i.e. grades IV-V, with normal kidneys; and reflux grades (II-)III-V, with scarred kidneys or ureteral anomalies). Refluxing primary megaureter is the only contraindication to endoscopic treatment. For reflux grades I-II and normal kidneys, no treatment is necessary, although endoscopic treatment may be an option for those with reflux grade II and bladder dysfunction. Children with grade III reflux and normal kidneys may be considered for treatment, although nontreatment with careful monitoring may also be an option for this group. Open surgery is reserved for use only in the ^∼^10% of children not responding to endoscopic treatment, and patients with refluxing primary megaureter. To support this approach, a new VUR classification system would be required, the aim being to ensure that we treat the patient and not the X-ray image from VCUG examination. In all cases, there is a need for thorough discussion of the treatment options with the parents of children with VUR and, if they are old enough, the patient as well.

Little investigation of patients is required after endoscopic treatment, provided that cure is demonstrated by a single VCUG performed 6 weeks to 3 months posttreatment. The only further check would be a dimercaptosuccinic acid (DMSA) scan after 12 months.

## CONCLUSIONS

Endoscopic treatment is clearly beneficial for patients with VUR: it provides a convenient means of curing the condition through a single procedure, without the need for major surgery. The cure rates with NASHA/Dx gel approach those seen with open surgery, but with a lower incidence of posttreatment UTI. As a result, the need for open surgery is now limited (although, importantly, endoscopic treatment with NASHA/Dx gel does not preclude subsequent open surgery). The use of endoscopic treatment is consistent with minimizing chronic use of antibiotics, and minimizing children's exposure to radiation. A revised approach to the management of VUR may now be considered, with patients classified according to several factors in addition to reflux grade. Further randomized, prospective studies are required to confirm the optimal management approach for VUR.
